# Phase I/pharmacokinetic/biochemical study of the nitroimadazole hypoxic cell sensitiser SR2508 (etanidazole) in combination with cyclophosphamide.

**DOI:** 10.1038/bjc.1993.424

**Published:** 1993-10

**Authors:** P. J. O'Dwyer, F. P. LaCreta, J. Walczak, T. Cox, S. Litwin, J. P. Hoffman, M. Zimny, R. L. Comis

**Affiliations:** Fox Chase Cancer Center, Philadelphia, Pennsylvania 19111.

## Abstract

SR2508 sensitises certain hypoxic tumor cells in vitro and in vivo to the cytotoxic action of radiation and alkylating agents. The mechanism of sensitisation may derive in part from depletion of glutathione (GSH) and possibly inhibition of GSH-dependent enzymes in target cells. We treated 46 evaluable patients with cyclophosphamide 750-1000 mg m-2 followed by SR2508 at eight dose levels ranging from 2.5 to 15.0 g m-2. Each patient received SR2508 as a single agent initially, followed a week later by the combination of cyclophosphamide and SR2508. Initially, myelosuppression was the major toxicity; potentiation of cyclophosphamide-induced leukopenia by SR2508 required a dose reduction of cyclophosphamide to 750 mg m-2 at SR2508 doses above 7.2 g m-2. At doses above 9.4 g m-2 an acute syndrome of muscle pains and painful paresthesias of the extremities lasting 12-24 h was observed to occur with increasing severity. This side-effect was intolerable in two of three patients treated at 15.0 g m-2. The only other reproducible side-effect was nausea and vomiting which was controllable with antiemetics. Plasma and urine SR2508 concentrations were measured by HPLC in 45 patients. Plasma elimination curves fit a 2-compartment model. The mean terminal half-life at each dose level ranged from 5.1-5.8 h. The mean area under the plasma concentration-time curve was linearly related to dose, and mean total body clearance ranged from 46.6-94.0 ml-1 min-1 m-2; renal clearance accounted for 65.7-79.3%. Pretreatment with cyclophosphamide did not influence the kinetics of SR2508 in individual patients. Examination of the glutathione content of peripheral mononuclear cells and tumour samples showed that depletion to below 50% of control occurred in the majority of patients. GSH transferase inhibition occurred with a similar time-course, but to a lesser extent. These data suggest that the further evaluation of this regimen should be conducted with SR2508 administration preceding that of cyclophosphamide and that its evaluation in cyclophosphamide-sensitive tumours is warranted.


*Br. J. Cancer* (1993), **68**, 756–766 © Macmillan Press Ltd., 1993

### Phase I/pharmacokinetic/biochemical study of the nitroimadazole hypoxic cell sensitiser SR2508 (etanidazole) in combination with cyclophosphamide

P.J. O'Dwyer, F.P. LaCreta, J. Walczak, T. Cox, S. Litwin, J.P. Hoffman, M. Zimny & R.L. Comis

*Fox Chase Cancer Center, Philadelphia, Pennsylvania 19111, USA.*

**Summary** SR2508 sensitises certain hypoxic tumour cells *in vitro* and *in vivo* to the cytotoxic action of radiation and alkylating agents. The mechanism of sensitisation may derive in part from depletion of glutathione (GSH) and possibly inhibition of GSH-dependent enzymes in target cells. We treated 46 evaluable patients with cyclophosphamide 750–1000 mg $m^{-2}$ followed by SR2508 at eight dose levels ranging from 2.5 to 15.0 g $m^{-2}$. Each patient received SR2508 as a single agent initially, followed a week later by the combination of cyclophosphamide and SR2508. Initially, myelsuppression was the major toxicity; potentiation of cyclophosphamide-induced leukopenia by SR2508 required a dose reduction of cyclophosphamide to 750 mg $m^{-2}$ at SR2508 doses above 7.2 g $m^{-2}$. At doses above 9.4 g $m^{-2}$ an acute syndrome of muscle pains and painful paresthesias of the extremities lasting 12–24 h was observed to occur with increasing severity. This side-effect was intolerable in two of three patients treated at 15.0 g $m^{-2}$. The only other reproducible side-effect was nausea and vomiting which was controllable with antiemetics. Plasma and urine SR2508 concentrations were measured by HPLC in 45 patients. Plasma elimination curves fit a 2-compartment model. The mean terminal half-life at each dose level ranged from 5.1–5.8 h. The mean area under the plasma concentration-time curve was linearly related to dose, and mean total body clearance ranged from 46.6–94.0 $ml^{-1}$ $min^{-1}$ $m^{-2}$; renal clearance accounted for 65.7–79.3%. Pretreatment with cyclophosphamide did not influence the kinetics of SR2508 in individual patients. Examination of the glutathione content of peripheral mononuclear cells and tumour samples showed that depletion to below 50% of control occurred in the majority of patients. GSH transferase inhibition occurred with a similar time-course, but to a lesser extent. These data suggest that the further evaluation of this regimen should be conducted with SR2508 administration preceding that of cyclophosphamide and that its evaluation in cyclophosphamide-sensitive tumours is warranted.

Resistance of the common slow-growing solid tumours to current chemotherapeutic strategies appears in part to be a consequence of the abnormal metabolic environment of solid tumours. It is proposed that the disordered vascular development in the growing tumor mass results in a proportion of cells being remote from capillaries; the increased diffusion distance leads of hypoxia, low concentrations of glucose and other nutrients, and reduced cellular pH (Thomlinson & Gray, 1955). Hypoxic cells are resistant both to radiation and to electrophilic cytotoxic drugs (Sutherland, 1988; Crabtree & Cramer, 1933). The proportion of hypoxic cells in a tumour is in part a function of tumour size, but even small tumours (1 mm in diameter) may have radiobiologically-defined hypoxic fractions ranging from 10–30%. The tumour types in which significant hypoxic fractions have been identified include all of the common solid tumours especially lung, colon, head and neck and breast cancers (Rockwell & Moulder, 1990; Adams & Stratford, 1986).

SR2508 (etanidazole, NSC 301467) is a 2-nitroimidazole, one of a series of 'oxygen mimetic' drugs which have been evaluated recently in clinical and preclinical studies (reviewed in Adams & Stratford, 1986, and Coleman *et al.*, 1988). In addition to sensitising hypoxic cells to radiation, the 2-nitroimidazoles restore the sensitivity of hypoxic cells to cytotoxic drugs *in vitro* and *in vivo*. This effect was first demonstrated by Clement *et al.*, 1980 and Rose *et al.*, 1980 and is particularly marked for alkylating agents. Misonidazole increased the antitumour activity of melphalan and of cyclophosphamide by factors of 2.2 and 1.8 respectively, while myelotoxicity was increased only by 1.2 and 1.3-fold (Law *et al.*, 1981; Hirst *et al.*, 1984). At equimolar doses, SR2508 enhances the activity of cyclophosphamide and melphalan as effectively as misonidazole (Hirst *et al.*, 1984). SR2508 was synthesised as a less lipophilic congener of misonidazole, since the neurotoxicity of nitroimidazoles is directly related to their lipophilicity, and misonidazole produced neurotoxicity at doses that yielded plasma concentrations insufficient for sensitisation (Brown & Workman, 1980). In the preclinical *in vivo* study of Clement and colleagues, the maximum sensitising efficacy increased both with the dose of cyclophosphamide and that of the nitroimidazole (Clement *et al.*, 1980). Therefore a determination of the maximum tolerated dose of SR2508 in a dose-escalating phase I study was undertaken.

Several mechanisms have been proposed to account for the chemosensitising effects of 2-nitroimidazoles (Mulcahy, 1986; Taylor *et al.*, 1984; Taylor *et al.*, 1983; Murray & Meyn, 1984, and Roizen-Towle *et al.*, 1984). Potentiation of alkylator-induced DNA damage has been identified through the use of alkaline elution (Taylor *et al.*, 1983). An inhibitory effect on DNA repair has been postulated but not proven (Taylor *et al.*, 1983). Others have suggested that an effect upon the pharmacokinetics of the alkylating agent may account for the sensitisation observed *in vivo* (Hinchcliffe *et al.*, 1983). The nitroimidazoles may themselves be cytotoxic to cells under conditions of hypoxia; drug treatment-associated DNA strand breaks have been described, and binding of labelled SR2508 to DNA is reported (Smith, 1984).

The early studies of 2-nitroimidazoles showed that they depleted cells of glutathione (GSH) (Varnes *et al.* pp. 121–126, 1990). Recent work has shown that GSH depletion may be an important means of reversing resistance to alkylating agents and platinum compounds (Ozols *et al.*, 1990). In addition, there is some evidence that 2-nitroimidazoles inhibit glutathione transferases, overexpression of which is associated with alkylating agent resistance (Kumar & Weiss, 1986, and Wang & Tew, 1985). Thus with the potential to address resistance on the basis of two distinct mechanisms, it was important to study further the effects of SR2508 on these pathways *in vivo*.

The initial clinical trials of SR2508 as a radiosensitiser

Correspondence: P.J. O'Dwyer, Fox Chase Cancer Center, 7701 Burholme Avenue, Philadelphia, Pennsylvania 19111, USA.
Received 12 February 1993; and in revised form 14 May 1993.

indicated that total doses substantially higher than those of misonidazole could be administered (Coleman *et al.*, 1986). These studies did not however characterise the maximum tolerated single dose of the sensitiser. The present study was designed to (1) establish the maximum tolerated dose of SR2508 in combination with cyclophosphamide; (2) identify acute dose-limiting toxicities; (3) describe the pharmacokinetics of SR2508 at these doses; and (4) determine the effects on GSH levels and GST activity in the peripheral mononuclear cells, red cells, and where possible, tumour cells of patients treated with these drugs.

The major issue in designing an initial regimen of SR2508 in combination with cyclophosphamide was the schedule of drug administration. Preclinical *in vivo* studies had produced conflicting results concerning the necessity for preincubation. Siemann and colleagues (1986) had shown that the optimal schedule appeared to be when the alkylating agent (CCNU, melphalan) and the sensitiser (misonidazole, SR 2508) were given close to the same time, even though in some tumours preincubation with a sensitiser was more effective. On the other hand, Clement *et al.*, 1980 showed that in the M5076 murine model, a two-fold increase in activity resulted when the misonidasole and cyclophosphamide were administered close to simultaneously. In the study conducted at Fox Chase Cancer Center, we elected to schedule the maximum plasma alkylating activity and maximum sensitiser levels at approximately the same time, and to perform biochemical measurements which could guide us to future schedule selection. Thus to allow for hepatic activation of cyclophosphamide, administration of the alkylator preceded that of SR2508 by 4 h. In addition, to describe the pharmacokinetics of SR2508 separately from cyclophosphamide, each patient received a single dose of SR2508 one week before the combination was administered.

### Materials and methods

### *Patient population*

Patients eligible for this study had a histologic diagnosis of cancer, and had exhausted the standard therapeutic options for their disease. They had recovered from all toxicity of prior treatment and had not had prior chemotherapy or radiation therapy within four weeks of entry to the study. They were aged $\geqslant 18$, and had an ECOG performance status of 0–2. They had adequate bone marrow (white cell count $\geqslant 3500$ cells $mm^{-3}$, platelets $\geqslant 100,000\ mm^{-3}$), liver (bilirubin $\leqslant 2\ mg\ dl^{-1}$), and kidney (creatinine $\leqslant 1.5\ mg\ dl^{-1}$) function. Patients with clinical evidence of peripheral neuropathy, or those who had prior treatment with vinca alkaloids, were not eligible. All patients gave written informed consent in accordance with Federal, State, and Institutional guidelines.

Before beginning therapy, a full history and physical examination, complete blood count, biochemical profile, urinalysis, electrocardiogram, and chest x-ray were performed. Patients were monitored with weekly blood counts and biochemical profiles, and clinical examinations on each course. Doses were reduced if necessary based on toxicity; doses were not escalated within patients. Results are reported using the consensus toxicity criteria (Cancer Therapy Evaluation Program, National Cancer Institute, Bethesda, Maryland, 1988). Patients with measurable disease were evaluated (usually by scan or x-ray) every other course; those with stable disease or better were continued on therapy. Response criteria were standard (Miller *et al.*, 1981).

### *Treatment plan*

SR2508 was supplied by the National Cancer Institute, Bethesda, Maryland, in 10 ml vials containing 1 gm of a white lyophilised powder. Vials were reconstituted with 0.9% sodium chloride, and further with either 5% dextrose injection, U.S.P. or 0.9% sodium chloride to yield a concentration no greater than $20\ mg\ ml^{-1}$. Higher concentrations were found to result in pain at the injection site radiating proximally along the vein. The appropriate volume was administered IV as a zero order infusion over 30 or 60 min. Drug administration and Pharmacokinetic sampling were performed in the Mary S. Schinagl Clinical Pharmacology Unit at Fox Chase Cancer Center.

### *Study design*

The design of the study called for an initial dose of SR2508 ($1\ gm\ m^{-2}$) to be administered one week before the combined treatment. The use of this lower dose allowed determination of both dose dependency and the effects of cyclophosphamide upon SR2508 pharmacokinetics, using the patient as their own control. One week later, cyclophosphamide $1\ gm\ m^{-2}$ was administered IV over 10 min, and exactly 4 h later, SR2508 as 30 min or 60 min infusion. The starting dose of SR2508 was $2.5\ gm\ m^{-2}$, a dose which had been tolerable in the phase I trial of SR2508 as a radiosensitiser. In subsequent courses, the same dose and schedule of SR2508 and cyclophosphamide were administered at three to four week intervals, without dose escalation in individual patients. Since definition of the cumulative maximum tolerated dose was of interest as part of this study, six patients were entered at each dose level.

Doses of SR2508 were escalated from 2.5 to $15\ gm\ m^{-2}$ in nine escalations. Following the accrual of 28 patients, a review of the pharmacokinetic data showed that the pharmacokinetics of SR2508 in individual patients were identical on weeks one and two. Accordingly, the design was changed to administer the same dose of SR2508 on weeks one and two. At $7.2\ gm\ m^{-2}$, myelosuppression with the combination of cyclophosphamide and SR2508 became dose-limiting. Since the myelosuppression was a reflection of the effect of SR2508 upon cyclophosphamide toxicity, and not solely a toxicity attributable to SR2508, the dose of cyclophosphamide was reduced by 25%, and escalation continued to the maximum tolerated dose of SR2508. The maximum tolerated dose was defined as the dose at which over 50% of patients had grade 3 or 4 toxicity attributable to SR2508.

### *Pharmacokinetic sampling*

Blood samples (5 ml) were drawn into Vacutainer tubes containing no anticoagulants, and centrifuged after 20 min. Serum was separated and stored at −40°C. Blood samples were obtained at baseline (before infusion), and at 0.25, 0.5, 1, 1.5, 2, 4, 8, 12, 24, 36, 48, and 72 h after drug administration. Urine was collected in four hour aliquots for 24 h, and in 12 h aliquots subsequently for a total of 48 h after each dose. Urine collections were kept on ice in the dark. Aliquots (5 ml) from each specimen were stored at −40°C for later analysis.

### *Analytical method*

SR2508 concentrations in plasma and urine were measured by the method of Workman *et al.*, pp.291–361, (1983). Briefly, samples were thawed and deproteinised with methanol, and the supernatant evaporated to dryness under a stream of $N_2$ at room temperature. The residue was resuspended in mobile phase and injected (by way of a Hewlett Packard automated injector) onto a $C_{18}$ Versapack column (30 × 4.1 mm ID) in a Hewlett Packard HP1090 HPLC system. The mobile phase was 5% methanol in water; SR2508 had a retention time of 4 min. Detection was by uv absorbance at 323 nm; peak heights were integrated and stored by a HP 85-B data system, and concentrations determined by reference to a standard curve which was run with each batch of samples. No other nitro-containing species have been detected in plasma, urine, or tissue samples using this analytic procedure. The day to day coefficient of variation (CV) less than 10% from 0.1 to $50\ \mu g\ ml^{-1}$, and the within-day CV was $<4\%$. The lower limit of detection was

$50\,ng\,ml^{-1}$, and the detector response was linear up to $50\,\mu g\,ml^{-1}$.

*Biochemical assays for GSH and GSH transferase*

GSH and GSH transferase were measured in peripheral mononuclear cells obtained from samples of whole blood (5 ml) layered over Ficoll-Hypaque and centrifuged at 400 *g* for 15–20 min. Blood samples were obtained at baseline, 1, 2, 4, 8, 12, 24 and 48 h after drug administation. In selected patients, tumour biopsies were obtained at baseline, 12 and 24 h following treatment. Biopsy samples were obtained by surgical excision, and the tissue was stored at – 70°C. At the time of assay, red cells, peripheral mononuclear cells, and tissue samples were thawed, resuspended in phosphate buffered saline, and homogenised using a Polytron (for tissue samples) or sonication (for red blood cell and peripheral mononuclear cell samples). GSH transferase was assayed spectrophotometrically by measuring the formation of a conjugate of glutathione and 1-chloro-2,4-dinitrobenzene (CDNB) as described by Habig and Jakoby, 1981. GSH was measured by a modification of the method of Griffith *et al.*, in which the rate of formation of a GSH conjugate of 5,5′-dithio-bis (2-nitrobenzoic acid) (DTNB) is determined spectrophotometrically (Griffith, 1980). Observed GSH and GSH transferase levels were normalised to protein content using the Bradford assay Bio-Rad, Richmond, VA).

*Data analysis*

Pharmacokinetic analysis of each patient's plasma samples was carried out using NONLIN84 (Statistical Associates, Lexington, Kentucky). For SR2508, the plasma levels best fit a two-compartment open model, represented by the equation:

$$C_t = Ae^{-\alpha t} + Be^{-\beta t}$$

incorporating a correction factor for length of infusion. Sample weighting of $1/y^2$ was used throughout. The area under the plasma concentration-time curve was obtained by integration of the fitted equation. Clearance and volume parameters were calculated by standard methods (Gibaldi & Perrier, 1982).

The pronounced inter-patient variability of biochemical results led to difficulty in applying standard regression analysis methods to the GSH and GSH transferase data. Because of the lack of a clear dose-response relationship, the pooled data from all available patients were analysed separately for both the first and second courses to allow an assessment of the extent and time-course of the observed changes. The data are described in terms of median effects as a function of time after SR2508 dosing. Comparisons of values of GSH and GSH transferase at all time points were made with baseline values using the Wilcoxon 2-sample test (two-tailed). We did not attempt to account for the many tests completed on the data. Evidence for relationships between various clinical, pharmacokinetic, and biochemical parameters were sought using Spearman's rank correlation test.

### Results

Forty-seven patients received 96 courses (range 1 to 11, median 2) of cyclophosphamide in combination with SR2508 between March 1987 and May 1989. The dose-escalation schema as modified by clinical and pharmacologic results is shown (Table I). At $7.2\,g\,m^{-2}$ of SR2508 and $1\,g\,m^{-2}$ cyclophosphamide, dose-limiting myelosuppression required a 25% reduction in the dose of the latter. At this level too, analysis of the pharmacokinetic results to that time showed no evidence of dose-dependency; accordingly the dose on the first week (without cyclophosphamide) was increased to equal that given with the alkylator.

The demographic characteristics of patients treated on this study are shown (Table II). Eighty-five per cent of the patients were minimally symptomatic. Most of the patients were heavily pretreated and had diseases that are generally refractory to alkylating agents. Patients received a median of two cycles of treatment (range 1–11).

**Table I** Phase I Study SR2508/cyclophosphamide: dose levels

| *Level* | *Week 1 SR2508* | *Week 2 Cyclophosphamide 2 dose* | *Week 2 SR2508 dose* | *Number of patients* |
|---|---|---|---|---|
| 1 | 1 | 1000 | 2.5 | 6 |
| 2 | 1 | 1000 | 3.25 | 6 |
| 3 | 1 | 1000 | .4 | 6 |
| 4 | 1 | 1000 | 5.5 | 6 |
| 5 | 1 | 1000 | 7.2 | 3 |
| 6 | 7.2 | 1000 | 7.2 | 3 |
| 7 | 9.4 | 750 | 9.4 | 6 |
| 8 | 12.2 | 750 | 12.2 | 5 |
| 9 | 15 | 750 | 15. | 3 |

**Table II** Demographic characteristics of the study population

| | | |
|---|---|---|
| Patients entered/evaluable | | 47/46 |
| Male/female | | 28/19 |
| median age (range) | | 60 (35–76) |
| Performance status | 0 | 14 |
| | 1 | 26 |
| | 2 | 7 |
| *Prior therapy* | | |
| Chemotherapy | | 17 |
| Chemotherapy + radiation | | 21 |
| Biologicals + chemotherapy | | 4 |
| Biologicals + radiation | | 1 |
| No prior treatment | | 4 |
| *Tumour type* | | |
| Colon | 24 | |
| Lung | 8 | |
| Ovarian | 2 | |
| Other | 13 | |

*Myelosuppression*

As expected, a major toxicity of treatment with SR2508 and cyclophosphamide was myelosuppression. Nadir counts were usually observed on d14 with recovery by d21, and neutrophils were affected almost exclusively: thrombocytopenia was sporadic. These characteristics implicate cyclophosphamide as the major contributor to this toxicity. Unexpectedly, the severity of leukopenia and neutropenia was related to the dose of SR2508: at the doses at which cyclophosphamide was administered at $1\,gm^{-2}$ there is a clear increase in the grade of leukopenia with increasing SR2508 dose in the range 2.5 to $7.2\,g\,m^{-2}$ (Table III). At higher SR2508 doses, with cyclophosphamide $750\,mg\,m^{-2}$, myelosuppression was tolerable. When neutropenia was analysed as the per cent decrease in neutrophil count (i.e. [pretreatment-nadir]/pretreatment) it was strongly correlated with SR2508 dose ($P<0.01$) at SR2508 doses up to $7.2\,g\,m^{-2}$ with cyclophosphamide $1000\,mg\,m^{-2}$ (Figure 1). This correlation was less pronounced at cyclophosphamide doses of $750\,mg\,m^{-2}$

**Table III** Neutropenia following SR2508 and cyclophosphamide administration

| *SR2508 dose ($g\,m^{-2}$)* | *Cyclophosphamide dose ($mg\,m^{-2}$)* | *Number of patients* | *Neutropenia–grade* 0 | 1 | 2 | 3 | 4 |
|---|---|---|---|---|---|---|---|
| 2.5 | 1000 | 6 | 4 | 0 | 0 | 1 | 1 |
| 3.25 | 1000 | 6 | 1 | 0 | 3 | 2 | 0 |
| 4.2 | 1000 | 6 | 2 | 0 | 1 | 1 | 2 |
| 5.5 | 1000 | 6 | 2 | 0 | 1 | 0 | 3 |
| 7.2 | 1000 | 7 | 1 | 0 | 0 | 3 | 3 |
| 9.4 | 750 | 6 | 2 | 1 | 0 | 1 | 3 |
| 12.2 | 750 | 6 | 1 | 0 | 1 | 1 | 3 |
| 15 | 750 | 3 | 2 | 0 | 0 | 1 | 0 |

**Figure 1** Relationship between the per cent decrease in neutrophil count ([pretreatment-nadir]/pretreatment) and SR2508 dose in patients treated with SR2508 and cyclophosphamide 1 gm m$^{-2}$.

and higher SR2508 doses. The per cent decrease in platelet count (not a clinically significant toxic effect) was not detectably related to SR2508 dose.

Doses of cyclophosphamide were not generally reduced in the face of myelosuppression unless recovery was particularly delayed. In four patients with grade 3 or 4 neutropenia, a 50% dose reduction ameliorated toxicity on subsequent courses. Of a total of 19 patients who developed grade 4 neutropenia, there were only three episodes of sepsis: one of these patients who also had Grade 4 thrombocytopenia, developed a fever, fell at home, and died of an apparent intracerebral hemorrhage. The other two patients recovered uneventfully.

Among eight patients who received three or more courses of SR2508 and cyclophosphamide, there was no evidence of cumulative myelosuppression.

*Neurotoxicity*

While myelosuppression was largely attributed to cyclophosphamide with a possible contribution of SR2508, the most important and dose-limiting toxicity attributable to SR2508 itself was neurotoxicity. From previous work with misonidazole, it was anticipated that central neurotoxicity (confusion, hallucinations, etc) might limit the *single* dose of SR2508. Peripheral neurotoxicity, manifest as a sensory motor neuropathy, was expected to limit the *cumulative* dose of SR2508. However the predominant neurologic manifestation was a novel sensory neuropathy in which a constellation of symptoms followed SR2508 administration as a single agent or in combination with the alkylating agent. At doses $\geqslant 9.4$ g m$^{-2}$, a syndrome of acute pain and tingling, radiating symmetrically down the extremities from the hip or shoulder girdles began from four to twelve hours after treatment. The severity of this paraesthesia-dysaesthesia pain syndrome was dose-related and was dose-limiting at 15 g m$^{-2}$ (Table IV). At this dose, two of three patients required high doses of morphine for pain relief. Resolution of symptoms occurred within 12 to 18 h, leaving no long term sequelae; subsequent sensory examinations were normal. The severity of these symptoms appeared to diminish with repeated dosing.

There was no evidence of central neurotoxicity at any dose studied in this trial. At doses higher than 7.2 g m$^{-2}$, patients were evaluated before, and at various times after SR2508 administration using the 'mini-mental' test of cortical function (Folstein *et al.*, 1975). Patients' scores did not change in the 24 h after treatment. Six patients experienced anxiety and/or depression in the weeks after treatment.

Cumulative neurotoxicity is more difficult to assess in a Phase I study, since patients do not usually receive a large number of courses. In the Radiation Therapy Oncology Group studies, considerable heterogeneity among patients is reflected in the maximum tolerated total dose of SR2508 which ranged from 21 to 40.8 g m$^{-2}$ (Varnes *et al.*, 1990). In this study 15 patients were treated with cumulative doses of SR2508 $\geqslant 21$ g m$^{-2}$. The maximum total dose administered was 60 g m$^{-2}$. Table V shows the incidence of peripheral neuropathy (vincristine-like chronic sensory impairment, as opposed to the acute phenomenon observed within hours of SR2508 administration) among groups of these patients. It seems clear that patients tolerate a total dose of up to 40 g m$^{-2}$ without difficulty. Small numbers at higher doses preclude a confident prediction of the cumulative maximum tolerated dose. This should be explored further in Phase II trials.

*Other toxicity*

Thirty-one of the 47 patients (66%) had nausea and vomiting following SR2508 administration. In all but three, symptoms were easily controlled with standard antiemetics, and treatment was only required during the 24 h following drug administration. In three patients, grade 3 vomiting prevented adequate oral intake: in these patients too, the symptoms resolved by 24 h.

Mild to moderate hepatic toxicity (as evidenced by increased liver enzymes or bilirubin) was also identified in 13 patients (28%). Many of those affected had liver involvement with tumour. Neither the nausea/vomiting nor the hepatic toxicity were dose-related.

*Pharmacokinetics*

Full pharmacokinetic studies were performed with and without cyclophosphamide in each patient. A typical plasma concentration-time curve is shown (Figure 2). The mean pharmacokinetic parameters at SR2508 doses from 2.5 to 7.2 g m$^{-2}$ are presented (Table VI). These patients received an initial SR2508 dose of 1 g m$^{-2}$. In all patients, the plasma concentration-time curve fit a two-compartment open model. The harmonic mean terminal half-life was remarkably reproducible among patients, varying from 5.1 to 5.8 h. The area under the concentration time-curve was linearly correlated with dose ($r = 0.88$, $P = 0.0001$) (Figure 3).

Since the sensitising efficacy of SR2508 depends upon its penetration of tissue compartments, we sought an estimate of overall tissue concentrations by considering the peripheral compartment represented in the kinetic model. Figure 4

**Table IV** Paresthesia-dysesthesia syndrome following SR2508 and cyclophosphamide administration

| *SR2508 dose (g m$^{-2}$)* | *Number of patients* | *Acute neurotoxicity–grade* 0 | 1 | 2 | 3 | 4 |
|---|---|---|---|---|---|---|
| 2.5 | 6 | 3 | 2 | 1 | 0 | 0 |
| 3.25 | 6 | 5 | 0 | 1 | 0 | 0 |
| 4.2 | 6 | 4 | 1 | 1 | 0 | 0 |
| 5.5 | 6 | 3 | 1 | 2 | 0 | 0 |
| 7.2 | 7 | 7 | 0 | 0 | 0 | 0 |
| 9.4 | 6 | 2 | 1 | 3 | 0 | 0 |
| 12.2 | 5 | 0 | 2 | 3 | 0 | 0 |
| 15.0 | 3 | 0 | 0 | 1 | 2 | 0 |

**Table V** Cumulative vincristine-like neuropathy associated with SR2508 administration

| *Total dose (g m$^{-2}$)* | *No. of patients* | *Grade* 0 | 1 | 2 | 3 |
|---|---|---|---|---|---|
| 0–10 | 22 | 19 | 2 | 1 | – |
| 11–20 | 10 | 8 | 2 | – | – |
| 21–30 | 6 | 1 | 2 | 1 | 2 |
| 31–40 | 4 | 2 | 2 | – | – |
| 41–50 | 4 | 2 | 1 | – | 1 |
| 51–60 | 2 | – | – | – | 2 |

**Table VI** Pharmacokinetics of SR2508 alone and in combination with 1000 mg m$^{-2}$ cyclophosphamide

| | Level I | | Level II | | Level III | | Level IV | | Level V | | Level Va[d] | |
|---|---|---|---|---|---|---|---|---|---|---|---|---|
| | *Alone*[a] | *2.5 g m$^{-2}$* | *Alone* | *3.25 g m$^{-2}$* | *Alone* | *4.2 g m$^{-2}$* | *Alone* | *5.5 g m$^{-2}$* | *Alone* | *7.2 g m$^{-2}$* | *Alone* | *7.2 g m$^{-2}$* |
| *n* | 6 | 6 | 6 | 6 | 6 | 6 | 6 | 6 | 4 | 4 | 3 | 3 |
| *Serum parameters* | | | | | | | | | | | | |
| AUC, μg × h ml$^{-1}$ | 292 ± 54[b] | 786 ± 152 | 311 ± 27.9 | 878 ± 79.9 | 185 ± 14.2 | 833 ± 105 | 335 ± 29.6 | 2047 ± 183 | 353 ± 5.1 | 2489 ± 169 | 1856 ± 163 | 1849 ± 141 |
| t1/2α, min[c] | 23.2 (16.1–34.9) | 15.5 (10.1–35.6) | 30.8 (20.4–41.6) | 27.8 (20.5–40.4) | 23.9 (17.5–34.6) | 22.5 (15.9–27.1) | 31.0 (25.0–37.0) | 17.6 (13.8–26.3) | 21.4 (11.4–33.4) | 20.4 (17.5–31.5) | 17.5 (11.7–27.0) | 17.8 (14.0–28.7) |
| t1/2β, h[c] | 4.74 (3.74–7.34) | 4.86 (3.97–6.50) | 5.56 (5.10–6.39) | 4.99 (4.12–5.87) | 4.65 (3.21 ± 5.92) | 4.92 (4.88–9.72) | 5.96 (6.75 ± 8.27) | 5.63 (4.46–6.77) | 5.93 (4.64–7.57) | 5.67 (5.07–6.40) | 4.24 (3.62–5.31) | 4.62 (4.06–5.20) |
| $Cl_{tot}$ ml min$^{-1}$ m$^{-2}$ | 63.4 ± 7.10 | 61.3 ± 9.06 | 55.5 ± 4.36 | 64.2 ± 5.59 | 92.6 ± 6.94 | 94.0 ± 16.5 | 51.4 ± 3.84 | 46.6 ± 4.03 | 47.3 ± 0.69 | 48.9 ± 3.26 | 67.4 ± 5.25 | 67.0 ± 4.53 |
| *Urine parameters* | | | | | | | | | | | | |
| $Cl_{renal}$, ml min m$^{-2}$ | 43.0 ± 5.89 | 45.8 ± 8.07 | 36.9 ± 3.81 | 48.1 ± 5.98 | 59.8 ± 6.09 | 61.8 ± 7.45 | 33.2 ± 3.56 | 36.9 ± 4.34 | 30.9 ± 1.53 | 37.8 ± 2.29 | 52.2 ± 5.30 | 47.9 ± 3.93 |
| % $Cl_{tot}$ | 67.8 | 74.7 | 66.5 | 74.9 | 64.6 | 65.7 | 64.6 | 79.2 | 65.3 | 77.0 | 77.5 | 71.5 |
| *Volumes of distribution* | | | | | | | | | | | | |
| $V_c$, l m$^{-2}$ | 9.44 ± 1.46 | 7.65 ± 0.73 | 9.00 ± 0.69 | 9.47 ± 1.12 | 14.4 ± 2.92 | 11.9 ± 1.65 | 10.7 ± 1.21 | 7.15 ± 0.97 | 8.26 ± 1.22 | 8.22 ± 0.66 | 8.75 ± 0.41 | 8.31 ± 0.61 |
| $V_{ss}$, l m$^{-2}$ | 21.6 ± 1.87 | 21.4 ± 1.33 | 21.6 ± 1.39 | 22.2 ± 0.91 | 31.9 ± 4.40 | 32.3 ± 3.87 | 23.0 ± 2.05 | 20.0 ± 1.83 | 21.3 ± 1.98 | 20.9 ± 0.59 | 21.0 ± 0.34 | 22.2 ± 0.67 |
| $V_\beta$, l m$^{-2}$ | 25.3 ± 2.09 | 25.2 ± 2.17 | 26.8 ± 1.90 | 27.6 ± 0.93 | 38.4 ± 4.08 | 40.4 ± 4.78 | 26.8 ± 2.05 | 23.2 ± 2.36 | 25.1 ± 2.68 | 24.0 ± 0.78 | 25.0 ± 0.54 | 26.7 ± 1.28 |

[a]SR2508 dose fixed at 1 g m$^{-2}$ when given alone in Levels I–V. [b]Values are mean ± s.e.m. [c]Values for t1/2α and t1/2β are harmonic mean (range). [d]SR2508 dose was identical given alone or in combination with 1 g m$^{-2}$ CTX dose level Va only.

**Figure 2** Plasma concentration time-curve from a patient treated with SR2508 9.4 g m$^{-2}$.

**Figure 3** Relationship of dose and area under the concentration-time curve among 47 patients treated with SR2508.

**Figure 4** Simulation of SR2508 concentration in plasma (central compartment, solid line) and tissue (peripheral compartment, dashed line) following a 5.5 g m$^{-2}$ dose. Concentrations were simulated using mean values of the first-order transfer and elimination constants obtained from non-linear regression analysis using a two-compartment body model of six patients at this dose level.

**Table VII** Pharmacokinetics of SR2508 alone and in combination with 750 mg $m^{-2}$ cyclophosphamide

| | *Level VI 9.36 g $m^{-2}$* | | *Level VII 12.2 g $m^{-2}$* | | *Level VIII 15 g $m^{-2}$* | |
|---|---|---|---|---|---|---|
| | *Alone* | *+ CTX* | *Alone* | *+ CTX* | *Alone* | *+ CTX* |
| *n* | 5 | 5 | 6 | 6 | 3 | 3 |
| *Serum parameters* | | | | | | |
| AUC, μg × h $ml^{-1}$ | 3933 ± 457[a] | 3397 ± 448 | 3541 ± 359 | 3372 ± 386 | 5003 ± 214 | 4114 ± 165 |
| t1/2α, min | 13.0[b] | 21.8 | 22.0 | 19.3 | 21.2 | 18.3 |
| | (7.43–21.7) | (16.5–27.5) | (15.5–28.7) | (14.0–23.3) | (12.8–38.6) | (16.2–22.7) |
| t1/2β, h | 5.61[b] | 5.53 | 4.30 | 4.24 | 4.71 | 4.68 |
| | (4.10–7.72) | (3.65–7.66) | (3.57–5.21) | (3.71–5.21) | (4.06–5.50) | (4.35–5.21) |
| $Cl_{tot}$ml $min^{-1}$ $m^{-2}$ | 42.2 ± 5.65 | 51.1 ± 10.0 | 60.4 ± 5.87 | 64.7 ± 7.84 | 48.8 ± 2.77 | 61.0 ± 2.36 |
| *Urine parameters* | | | | | | |
| $Cl_{renal}$, ml $min^{-1}$ $m^{-2}$ | 31.0 ± 3.94 | 39.2 ± 8.54 | 48.1 ± 6.48 | 57.0 ± 7.07 | 41.6 ± 0.74 | 44.1 ± 2.06 |
| % $Cl_{tot}$ | 73.5 | 76.7 | 79.6 | 88.1 | 85.3 | 72.3 |
| *Volumes of distribution* | | | | | | |
| $V_c$, l $m^{-2}$ | 6.61 ± 0.63 | 7.80 ± 0.91 | 8.01 ± 0.50 | 7.69 ± 0.50 | 8.19 ± 1.07 | 8.15 ± 0.43 |
| $V_{ss}$, l $m^{-2}$ | 18.6 ± 0.8 | 20.6 ± 0.7 | 18.7 ± 0.8 | 19.5 ± 1.1 | 17.6 ± 1.0 | 21.4 ± 0.6 |
| $V_β$, l $m^{-2}$ | 20.4 ± 0.9 | 23.9 ± 1.3 | 22.3 ± 1.1 | 23.4 ± 1.1 | 20.0 ± 1.1 | 24.8 ± 0.4 |

[a]Values are mean ± s.e.m. [b]Values are harmonic mean (range).

shows the model-predicted drug concentrations in the peripheral compartment. While these levels cannot be used to describe actual concentrations in a particular organ, they provide an estimate that may be of value in selecting doses for sensitising regimens. It may be seen that doses ⩾ 5.5 g $m^{-2}$ result in levels ⩾ 2 mM, a concentration sufficient to sensitise cells in many hypoxic systems (Brown & Workman, 1980).

Volumes of distribution were similarly reproducible among groups of patients. The volume of the central compartment approximated that of extracellular fluid, while the steady-state volume of distribution was consistent with drug distribution through the total body water, with minimal tissue binding.

The total-body clearance range from 46.6 to 94.0 ml $min^{-1}$ $m^{-2}$, and was not dose-related. Renal clearance accounted for approximately 70% of total body clearance, and about 70% of the dose was found in the urine in the 48 h collection period. Metabolites of SR2508 were not detected using this analytical method: no additional peaks were found on the chromatograms of serum or urine. The extent to which metabolism or hepatic excretion of SR2508 contributes to drug removal is unknown at this time but would be expected to account for the difference between total body clearance and renal clearance (30%).

The effect of cyclophosphamide on SR2508 pharmacokinetics may best be analysed in the patients who received the same dose with and without the alkylator (i.e. patients at 7.2, 9.4, 12.2 and 15 g $m^{-2}$). In Table VII are presented the principal pharmacokinetic parameters observed in these patients. Pretreatment with cyclophosphamide does not markedly influence the kinetics of SR2508.

**Figure 5** Total body clearance of SR2508 as a function of creatinine clearance.

The relationship between renal function and SR2508 disposition was analysed by plotting creatinine clearance (derived from a nomogram) against the total body clearance of SR2508 (Figure 5). As anticipated from the SR2508 urinary excretion data, the relationship was highly significant ($r = 0.626$, $P = .0001$).

Relationships between pharmacokinetic parameters and toxicity showed several positive correlations. The total body clearance of SR2508 (and therefore renal clearance) was significantly related to both white cell and neutrophil toxicity ($P < 0.005$) (Figure 6). Since this level of significance is much lower than that for either area under the concentration-time curve or SR2508 dose, it may be that the renal clearance of SR2508 and that of active metabolites of cyclophosphamide are related.

### *Biochemical results*

SR2508 at doses as low as 1 g $m^{-2}$ inhibits GSH transferase activity in peripheral mononuclear cells, but not in red cells (data not shown), by over 50%. The reason for this discrepancy is not clear, but may relate to the higher concentration of GSH in red blood cells. Depletion of peripheral mononuclear cell GSH levels parallels the inhibition of GSH transferase, which suggests a common mechanism.

The mean baseline value for GSH in peripheral mononuclear cells was 0.265 ( ± 0.037, SEM) $nmol^{-1}$ mg protein among 42 patients. When measured one week after the initial SR2508 dose, these values were unchanged at 0.254 ( ± 0.034, SEM) nmol $mg^{-1}$ protein. The baseline GSH transferase activity was 1.17 ( ± 0.14, SEM) nmol $min^{-1}$ $mg^{-1}$ protein among 43 patients. This value too was unchanged by SR2508 administration at 1.07 ( ± 0.15, SEM) nmol $min^{-1}$ $mg^{-1}$ protein in week 2.

Because of pronounced inter-patient variability, no dose-response relationship for GSH transferase inhibition or GSH depletion in peripheral mononuclear cells could be discerned. Visual inspection of the data showed that the values for both GSH and GSH transferase declined after SR2508 treatment, followed by recovery. Using the Wilcoxon two sample test (two-tailed), the values for GSH at 8 and 12 h were significantly different from those at other times ($P = .001$). The GSH transferase values at these times were also different from those at other times ($P = .004$).

To allow an assessment of the time-course of these effects, the data from all the patients were pooled. Figure 7 shows the median change in the values for the first and second courses respectively. Following the first course (SR2508 alone) 24% inhibition of GSH transferase activity compared to control was observed at 12 h, with recovery by 24 h. Following the second course (SR2508 and cyclophosphamide) a more profound effect (about 40% inhibition)

**Figure 6** Total body clearance of SR2508 with cyclophosphamide 1000 mg $m^{-2}$ *vs* leukopenia (Panel **a**) and neutropenia (Panel **b**) and total body clearance of SR2508 with cyclophosphamide 1000 mg $m^{-2}$ (**a** and **b**) and cyclophosphamide 750 mg $m^{-2}$ (**c** + **d**) *vs* leukopenia (**a** + **c**) and neutropenia (**b** + **d**). The percent decrease in blood counts is calculated from (pretreatment count-nadir count/pretreatment count) and expressed as a percentage.

reflects the contribution of both drugs, but with a similar time-course to that observed with SR2508 alone.

More marked effects were observed on the GSH content of peripheral mononuclear cells in this analysis. A decline in GSH concentration by 50% was found at 12 h following SR2508 treatment, with recovery by 24 h. Addition of cyclophosphamide did not result in a markedly greater degree of GSH depletion (61% of control). The changes in GSH transferase activity and GSH content at the foregoing but not other time-points were significantly different from baseline ($P<.05$).

There was no relationship between the observed biochemical changes in peripheral mononuclear cells and either clinical toxicity or pharmacokinetic parameters.

We obtained biopsies of tumour tissue before and at 12 and 24 h after SR2508 administration in three patients (Table VIII). Each received a dose of 9.4 to 12.2 g $m^{-2}$ of SR2508. The results of the GSH transferase and GSH analyses are concordant with the peripheral mononuclear cell data: pronounced interpatient variability is apparent. One patient had no evidence of a biochemical effect, while one had a modest and one a profound change in the measured activities. In these patients, pharmacokinetic differences did not explain the variability.

### Discussion

In this Phase I study, the maximum tolerated dose of SR2508 was sought based on preclinical evidence *in vitro* and *in vivo* that sensitising efficacy is a function of concentration or dose (Clement *et al.*, 1980; Brown *et al.*, 1981). Indeed, the failure of misonidazole to exhibit significant enhancement of alkylator efficacy has been attributed to an inability to yield a

**Table VIII** GSH and GSH transferase determination in tumour biopsies of patients treated with SR2508

| | *GSH transferase (% Control)* | | | *GSH (% Control)* | | |
|---|---|---|---|---|---|---|
| *Patient* | *IE* | *WM* | *AG* | *IE* | *WM* | *AG* |
| Baseline | 100 (46.7)[a] | 00(42.1) | 100(21.5) | 100(6.1) | 100(15.2) | 100(2.37) |
| 12 h | 129 | 59 | 21 | 143 | 51 | 20 |
| 24 h | 75 | 66 | 94 | 124 | 91 | 200 |

[a]Values in parentheses present control GSH transferase specific activity expressed as nmol $mg^{-1}$ protein $min^{-1}$ or GSH concentrations expressed as nmol $mg^{-1}$ protein.

clinically-important dose-modifying factor (Coleman *et al.*, 1988). (The dose-modifying factor is the ratio of doses or concentrations of a cytotoxic drug required to achieve a target effect in the presence *vs* the absence of the sensitising agent). Based on *in vitro* models, a dose-modifying factor of about 1.5 may be anticipated with SR2508 concentrations of $>2$ mM (428 μg ml$^{-1}$); our analysis of drug concentrations in the peripheral compartment suggests that such levels were achieved at doses $\geqslant 5.5$ g m$^{-2}$ in this study. Such doses would be expected to sensitise hypoxic cells to alkylating agents, and should be tested in Phase II trials. However, doses higher than 7.2 g m$^{-2}$ should probably not be used in regimens using conventional toxicity criteria, since potentiation of cyclophosphamide myelosuppression may require dose-reduction of the alkylating agent.

The maximum tolerated dose of a single dose of SR2508 was found to be 12.2 g m$^{-2}$. Above this, neurotoxicity was dose-limiting. The 'paraesthesia-dysesthesia syndrome' we have described appears to be unique to SR2508 among 2-nitroimidazoles, and is not described, to our knowledge, in response to other neurotoxins. The syndrome represents an acute, reversible sensory neuropathy. As with the biochemical effects, the onset of this syndrome was delayed, reaching a peak at 8–12 h following drug administration. This time

**Figure 7** Median (and upper and lower quartile) values for GSH content and GSH transferase activity in the peripheral mononuclear cells of patients treated with SR2508 alone (Cycle 1) (Panels **a** and **b**) and cyclophosphamide followed by SR2508 (Cycle 2) (Panels **c** and **d**).

course is consistent either with inhibition of intracellular processes eventually resulting in depletion of their product, or with the formation of a metabolite that may itself be neurotoxic.

SR2508 was chosen for development based on its low lipophilicity (Brown & Workman, 1980). As predicted, SR2508 causes little central neurotoxicity, though at many of the doses used in this study, mild psychological effects (anxiety, depression) were noted. Objective cortical impairment did not occur. The greater central effects reported with misonidazole suggest that differences in the rate of drug accumulation in the brain *vs* the peripheral nervous system may be responsible for this altered pattern of toxicity. Consistent with the delayed effects of SR2508 on the nervous system, the onset of nausea and vomiting (which occurred in the majority of patients) pursued a time course similar to that of the paraesthesia-dysesthesia syndrome. Similar findings have been reported in a Phase I trial of this combination from the University of Wisconsin (Bailey *et al.*, 1991).

Coleman and colleagues have demonstrated a relationship between the total area under the concentration-time curve of SR2508 and the probability of developing a chronic peripheral sensory neuropathy following repeated small doses of the drug (Coleman *et al.*, 1987). In the current study, the incidence of this type of sensory neuropathy associated with high cumulative doses of SR2508 was low. Total doses up to $60 \text{ g m}^{-2}$ were tolerated by two patients. These limited data suggest that the development of a peripheral sensory neuropathy may relate both to the AUC and to the schedule of administration: higher cumulative doses may be tolerated using intermittent schedules such as this. Future phase II studies of SR2508 will clarify the cumulative dose tolerance of the drug on this schedule, and describe its relationship to pharmacokinetic parameters.

The relationship of myelosuppression following combined treatment with SR2508 and cyclophosphamide to SR2508 dose, and more accurately, to SR2508 clearance, is of some interest. An obvious interpretation is that SR2508 (not a known myelotoxic agent) may potentiate the toxicity of cyclophosphamide to bone marrow stem cells. This may occur as a result of GSH depletion in oxic cells as occurred in peripheral mononuclear cells; enhanced toxicity of cyclophosphamide (Grau *et al.*, 1990) and other alkylators (Durand & Chaplin, 1987; Horsmann *et al.*, 1989) to oxic cells is well-described *in vivo* and in spheroids. Allalunis *et al.* have shown in addition that a proportion of normal marrow cells exist in a hypoxic environment (Allalunis *et al.*, 1983). Sensitisation of these cells by SR2508 may contribute to the enhancement of myelosuppression observed.

An alternative explanation is suggested by the pharmacokinetics. The close relationship between SR2508 and creatinine clearance may also exist between renal function and the plasma clearance of the active metabolites of cyclophosphamide. Thus, indirectly SR2508 clearance and clearance of alkylating species may be related. However, a potentiating effect of SR2508 at the level of the marrow is supported by the dose-related enhancement of marrow toxicity.

As others have reported, the pharmacokinetics of SR2508 are remarkably uniform among patients. Our results are almost identical to those of Bailey *et al.* (Bailey *et al.*, 1991). Our studies of SR2508 content in tumour biopsies confirm those of others showing that tumour drug levels are comparable to those of simultaneously obtained plasma levels (Coleman *et al.*, 1984). since sensitisation by SR2508 requires its intracellular metabolism, activities of the enzymes mediating one-electron reduction are potentially important determinants of drug effect. Individual variation in sensitising efficacy may thus depend upon the capacity of the hypoxic tumour tissue to metabolise SR2508 to its one-electron reduction intermediates. The nitroreductase activity of tumours was not characterised in this study, but may be an important determinant of and predictor for response.

Such variation may explain the biochemical findings in peripheral mononuclear cells. Inhibition of GSH transferase and depletion of GSH followed the administration of SR2508 in a majority of the patients. Effects were observed at the lowest doses of SR2508, but inter-patient variability was pronounced: in some patients no biochemical changes occurred. As a result, no clear relationship to dose could be established. Such a finding often indicates population variability in drug handling, but since the proportion of SR2508 eliminated non-renally is small (over 70% unchanged in the urine) a pharmacogenetic pattern is not evident from the pharmacokinetic data. Future studies will address the role of metabolic phenotype in determining the biochemical effects of SR2508.

The medians of the pooled data from the 47 patients showed a clear and significant trend to a nadir of GSH content and GSH transferase activity at 8–12 h after dosing. A similar, delayed depletion of GSH was observed in murine tumours and normal tissues following SR2508 administration i.p. (Horsmann *et al.*, 1989). In both species the time of drug administration varied throughout the day so that a circadian variation alone is unlikely to be responsible for the observed effects. The mechanism of the depletion of GSH is not known. A metabolite which has been identified *in vitro*, but not hitherto *in vivo*, in the GSH conjugate of SR2508 (Varghese & Whitmore, 1986). Our current studies are directed to the further investigation of this derivative (O'Dwyer *et al.*, 1992).

Finally, it was hoped that the time course of biochemical changes in tumour tissue would provide a guide to the scheduling of SR2508 with the alkylating agent. The three patients in whom biopsies were obtained sequentially show a pattern of heterogenity similar to that observed in the studies of peripheral mononuclear cells. Inhibition of GSH transferase and depletion of GSH at 12 h in two of the three patients, with recovery of 24 h (in GSH levels at least) suggest that maximal biochemical effects in tumour probably occur with a time course similar to those in peripheral mononuclear cells. The time course of the biochemical changes and of certain of the toxicities (e.g. nausea and vomiting, and paresthesia-dysesthesia) indicate that many of the effects of SR2508 are exerted 8–12 h after dosing. Since some of the preclinical *in vivo* models support pretreatment with the nitroimidazole, these data suggest to us that modification of the schedule of administration for Phase II trials will be appropriate. Unlike the schedule reported here or the simultaneous administration of these drugs in the study of Bailey *et al.*, 1991, a schedule of SR2508 and cyclophosphamide which would administer the sensitiser first, followed 4 h later by cyclophosphamide would effectively allow an 8 h interval between SR2508 administration and the appearance of maximum alkylating activity. It is this schedule that might best be tested in Phase II trials of SR2508 as a sensitiser of alkylating agents.

We are grateful for the superior secretarial skills of Karen Smith and Catherine Thompson, and the expert data management of Catherine Janus.

This work was supported by grant CA49820-01 from the National Cancer Institute, NIH.

### References

ADAMS, G.E. & STRATFORD, I.J. (1986). Hypoxia-mediated nitroheterocyclic drugs in the radio-and chemotherapy of cancer: an overview. *Biochem. Pharmacol.*, **35**, 71–76.

ALLALUNIS, M.J., CHAPMAN, J.D. & TURNER, A.R. (1983). Identification of a hypoxic population of bone marrow cells. *Int. J. Radiat. Oncol. Biol. Phys.*, **9**, 227–232.

BAILEY, H., MULCAHY, R.T., TUTSCH, K.D., ROZENTAL, J.M., ALBERTI, D., ARZOOMANIAN, R.Z., TOMBES, M.B., TRUMP, D.L. & WILDING, G. (1991). A Phase 1 study of SR2508 and cyclophosphamide administered by intravenous injection. *Cancer Res.*, **51**, 1099–1104.

BROWN, J.M. & WORKMAN, P. (1980). Partition coefficient as a guide to the development of radiosensitizers which are less toxic than misonidazole. *Radiat. Res.*, **82**, 171–190.

BROWN, J.M., YU, N.Y., BROWN, D.M. & LEE, W.W. (1981). SR2508: a 2-nitroimidazole amide which should be superior to misonidazole as a radiosensitizer for clinical use. *Int. J. Radiat. Oncol. Biol. Phys.*, **7**, 695–703.

CLEMENT, J.J., GORMAN, M.S., WODINSKY, I., CATANE, R. & JOHNSON, R.K. (1980). Enhancement of antitumor activity of alkylating agents by the radiation sensitizer misonidazole. *Cancer Res.*, **40**, 4165–4172.

COLEMAN, C.N., URTASUN, R.C., WASSERMAN, T.H., HANCOCK, S., HARRIS, J.W., HALSEY, J. & HIRST, V.K. (1984). Initial report of the Phase I trial of the hypoxic cell radiosensitizer SR-2508. *Int. J. Radiat. Oncol. Biol. Phys.*, **10**, 1749–1753.

COLEMAN, C.N., URTASUN, R.C., WASSERMAN, T.H., HALSEY, J., HIRST, V.R., HANCOCK, S. & PHILLIPS, T.L. (1986). Phase I trial of the hypoxic cell radiosensitizer SR2508: the results of the five to six week schedule. *Int. J. Radiat. Oncol. Biol. Phys.*, **12**, 1105-1108.

COLEMAN, C.N., HALSEY, J., COX, R.S., HIRST, V.K., BLASCHKE, T., HOWES, A.E., WASSERMAN, T.H., URTASUN, R.C., PAJAK, T., HANCOCK, S., PHILLIPS, T.L. & NOLL, L. (1987). Relationship between the neurotoxicity of the hypoxic cell radiosensitizer SR2508 and the pharmacokinetic profile. *Cancer Res.*, **47**, 319-322.

COLEMAN, C.N., BUMP, E.A. & KRAMER, R.A. (1988). Chemical modifiers of cancer treatment. *J. Clin. Oncol.*, **6**, 709-733.

CRABTREE, H.G. & CRAMER, W. (1933). The action of radium on cancer cells. II. Some factors determining the sensitivity of cancer cells to radium. *Proc. Royal. Soc.*, **113**, 238-250.

DURAND, R.E. & CHAPLIN, D.J. (1987). Chemosensitization by misonidazole in CCNU-treated spheroids and tumours. *Br. J. Cancer*, **56**, 103-109.

FOLSTEIN, M., FOLSTEIN, S. & MCHUGH, P. (1975). Mini-mental state: a practical method for grading the cognitive state of patients for the clinician. *J. Psychiat. Res.*, **12**, 189-198.

GIBALDI, M. & PERRIER, D. (1982). *Pharmocokinetics*, 2nd Edition. Marcel Dekker, New York.

GRAU, C., BENTZEN, S.M. & OVERGAARD, J. (1990). Cytotoxic effect of misonidazole and cyclophosphamide on aerobic and hypoxic cells in a C3H memory carcinoma *in vivo*. *Br. J. Cancer*, **61**, 61-64.

GRIFFITH, O.W. (1980). Determination of glutathione and glutathione disulfide using glutathione reductase and 2-vinylpyridine. *Anal. Biochem.*, **106**, 207-212.

HABIG, W.H. & JAKOBY, W.B. (1981). Glutathione-S-transferase: assays for differentiation of glutathione-S-transferases. *Methods in Enzymol.*, **77**, 398-405.

HINCHCLIFFE, M., MCNALLY, N.J. & STRATFORD, M.R.L. (1983). The effect of radiosensitizers on the pharmacokinetics of melphalan and cyclophosphamide in the mouse. *Br. J. Cancer*, **48**, 375-383.

HIRST, D.G., HAZLEHURST, J.L. & BROWN, J.M. (1984). Sensitization of normal and malignant tissues to cyclophosphamide by nitroimidazoles with different partition coefficients. *Br. J. Cancer*, **49**, 33-42.

HORSMANN, R., WOOD, P.J. & BROWN, J.M. (1989). Misonidazole chemosensitization of EMT6 spheroids to melphalan. *Radiother. Oncol.*, **15**, 103.

KUMAR, K.S. & WEISS, J.F. (1986). Inhibition of glutathione peroxidase and glutathione transferase in mouse liver by misonidazole. *Biochem. Pharmacol.*, **35**, 3143-3146.

LAW, M.P., HIRST, D.G. & BROWN, J.M. (1981). The enhancing effect of misonidazole on the response of the RIF-1 tumor to cyclophosphamide. *Br. J. Cancer*, **44**, 208-218.

MILLER, A.B., HOOGSTRATEN, B., STAQUET, M. & WINKLER, A. (1981). Reporting results of cancer treatment. *Cancer*, **47**, 207-214.

MULCAHY, R.T. (1986). Cross link formation and chemopotentiation of EMT-6/RO cells exposure to miso after CCNU treatment *in vitro*. *Int. J. Radiat. Oncol. Biol. Phys.*, **12**, 1389-1392.

MURRAY, D. & MEYN, R.E. (1984). Enhancement of the DNA cross link activity of nitrogen mustard by misonidazole and diethyl maleate in a mouse fibrosarcoma tumor *in vivo*. *Cancer Res.*, **44**, 91-96.

O'DWYER, P.J., PANTING, L., LACRETA, F.P. & CLAPPER, M. (1993). SR2508 pharmacokinetics and biochemical effects in tumor and normal tissues of scid mice bearing HT-29 colon adenocarcinoma. *Biochem. Pharmacol.* (in press).

OZOLS, R.F., O'DWYER, P.J., HAMILTON, T.C. & YOUNG, R.C. (1990). The role of glutathione in drug resistance. *Cancer Treat. Rev.*, **17** (Suppl A), 45-50.

ROCKWELL, S. & MOULDER, J.E. (1990). Hypoxic fractions of human tumors xenografted into mice: a review. *Int. J. Radiat. Oncol. Biol. Phys.*, **19**, 197-202.

ROIZEN-TOWLE, L., BIAGLOW, J.E., MELTZER, H.L. & VARNES, M.E. (1984). Factors associated with the preincubation effect of hypoxic cell sensitizers *in vitro* and their possible implications in chemosensitization. *Radiat. Res.*, **98**, 506-518.

ROSE, C.M., MILLAR, J.L., PEACOCK, J.H., PHELPS, T.A. & STEPHENS, T.C. (1980). Differential enhancement of melphalan cytotoxicity in tumor and normal tissue by misonidazole. In *Radiation Sensitizers: Their Use in Clinical Management of Cancer*. Luther Brady (ed) p. 250. Masson Publishing: New York.

SIEMANN, D.W. & MULCHAHY, R.T. (1986). Sensitization of cancer chemotherapeutic agents by nitroheterocycles. *Biochem. Pharmacol.*, **35**, 111-115.

SMITH, B.R. (1984). Hypoxia-enhanced reduction and covalent binding of [2-$^3$H]-misonidazole in the perfused rat liver. *Biochem. Pharmacol.*, **33**, 1379-1381.

SUTHERLAND, R.M. (1988). Cell and environment interactions in tumor microregions: the multicell spheroid model. *Science*, **240**, 177-184.

TAYLOR, Y.C., SAWYER, J., HSU, B. & BROWN, J.M. (1984). Mechanism of melphalan cross link enhancement by misonidazole pretreatment. *Int. J. Radiat. Oncol. Biol. Phys.*, **10**, 1603-1607.

TAYLOR, Y.C., EVANS, J.W. & BROWN, J.M. (1983). Mechanism of sensitization of chinese hamster ovary cells to melphalan by hypoxic treatment with misonidazole. *Cancer Res.*, **43**, 3175-3181.

THOMLINSON, R.H. & GRAY, L.H. (1955). The histological structure of some human lung cancers and the possible implications for radiotherapy. *Br. J. Cancer*, **9**, 539-549.

VARGHESE, S.J. & WHITMORE, G.F. (1986). Indentification of a reactive glutathione conjugate as a metabolite of SR2508 in CHO cells. *Int. J. Radiat. Oncol. Biol. Phys.*, **12**, 1223-1226.

VARNES, M.E., BIAGLOW, J.E., KOCH, C.J. & HALL, E.J. (1990). Depletion of non-protein thiols of hypoxic cells by misonidazole and metronidazole. In: *Radiation Sensitizers: Their Use in the Clinical Management of Cancer*. Brady, L.L. (ed.) pp. 121-126, Masson Publishing, New York, USA.

WANG, A.L. & TEW, K.D. (1985). Increased glutathione-S-transferase activity in a cell line with acquired resistance to nitrogen mustards. *Cancer Treat. Rep.*, **69**, 677-682.

WORKMAN, P. (1983). Pharmacokinetics of radiosensitizing agents. In *Pharmacokinetics of Anticancer Agents in Humans*. Ames, M.M., Powis, G. & Kovach, J.S. (eds) Vol. 99, pp. 291-361. Elsevier: Amsterdam.


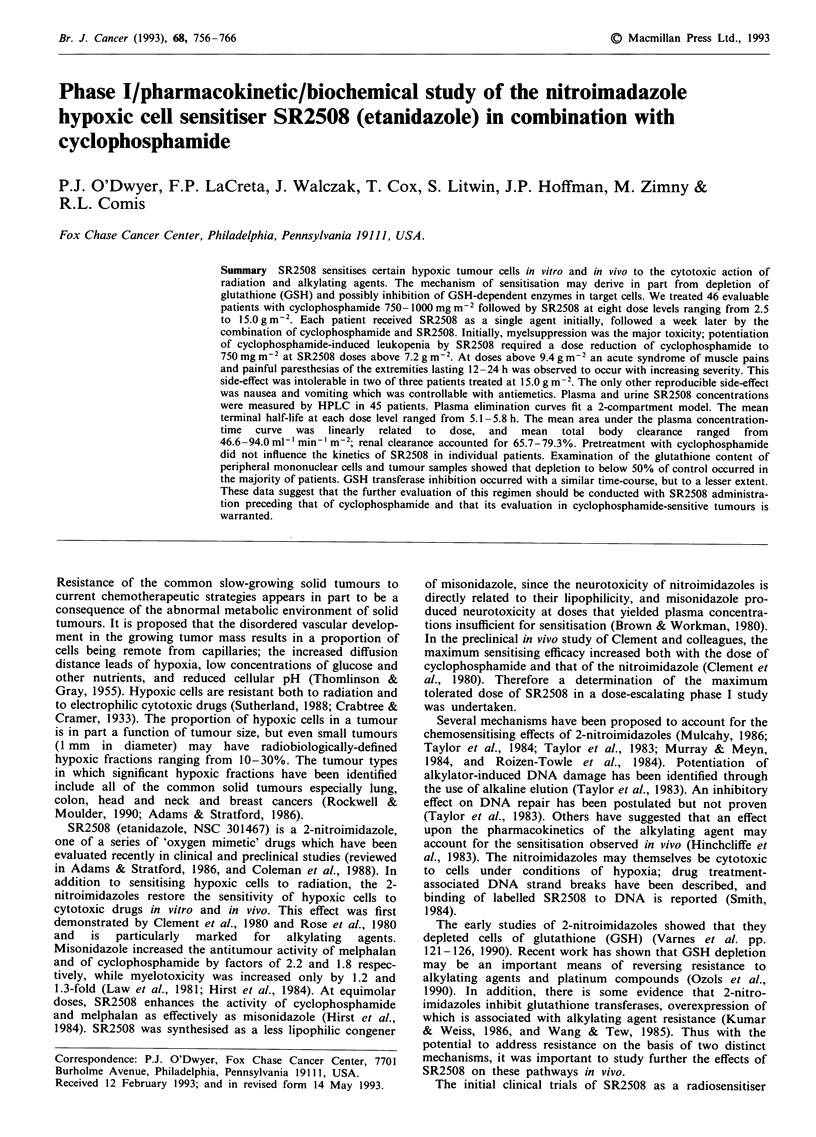

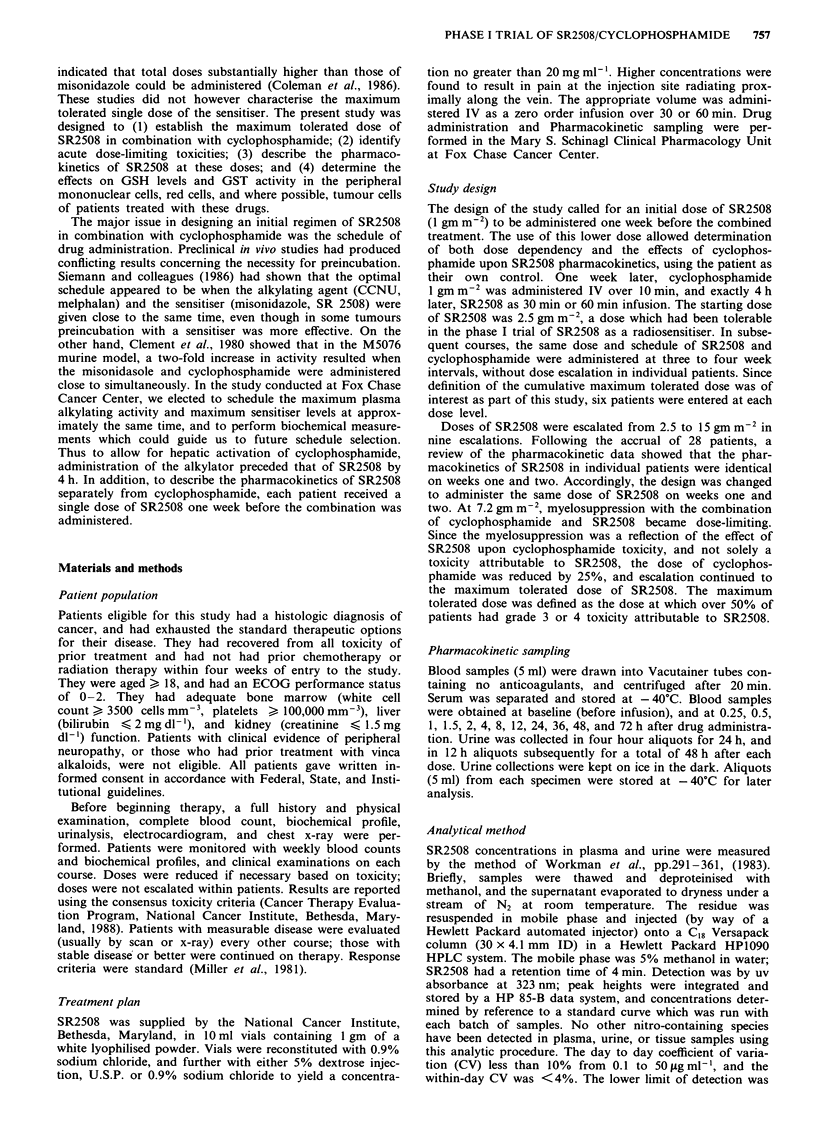

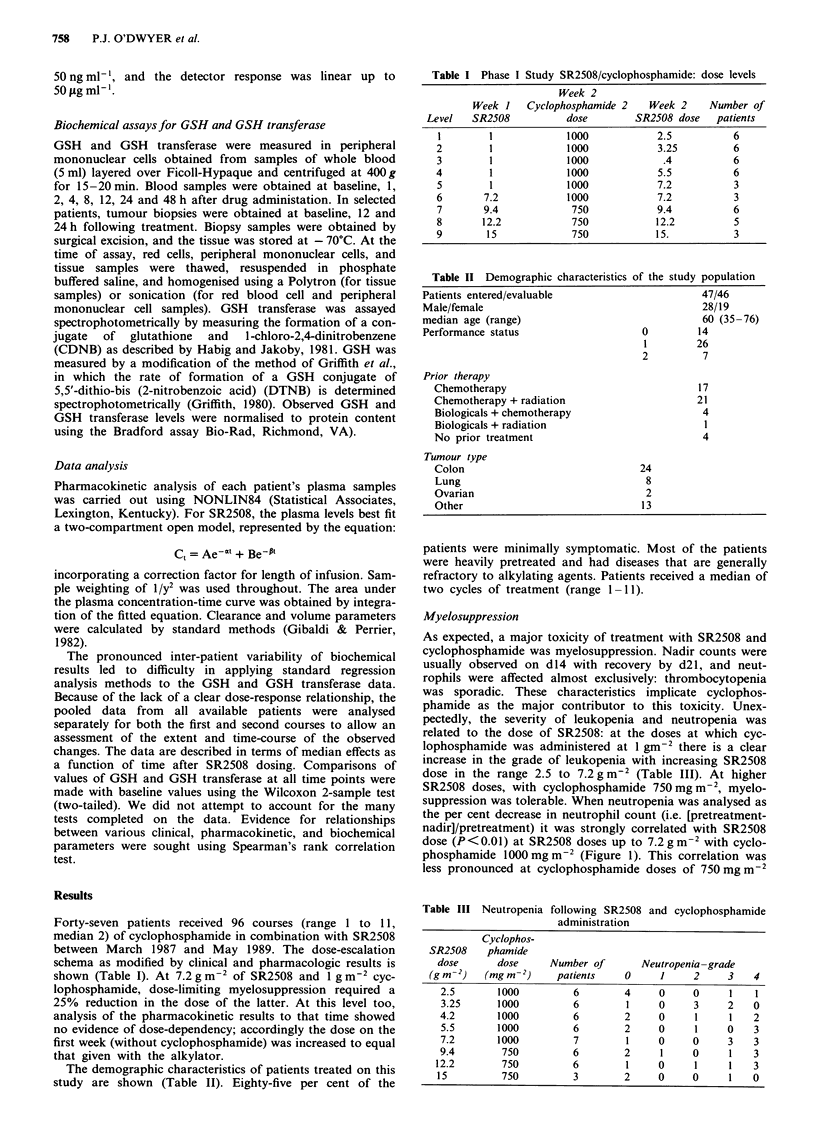

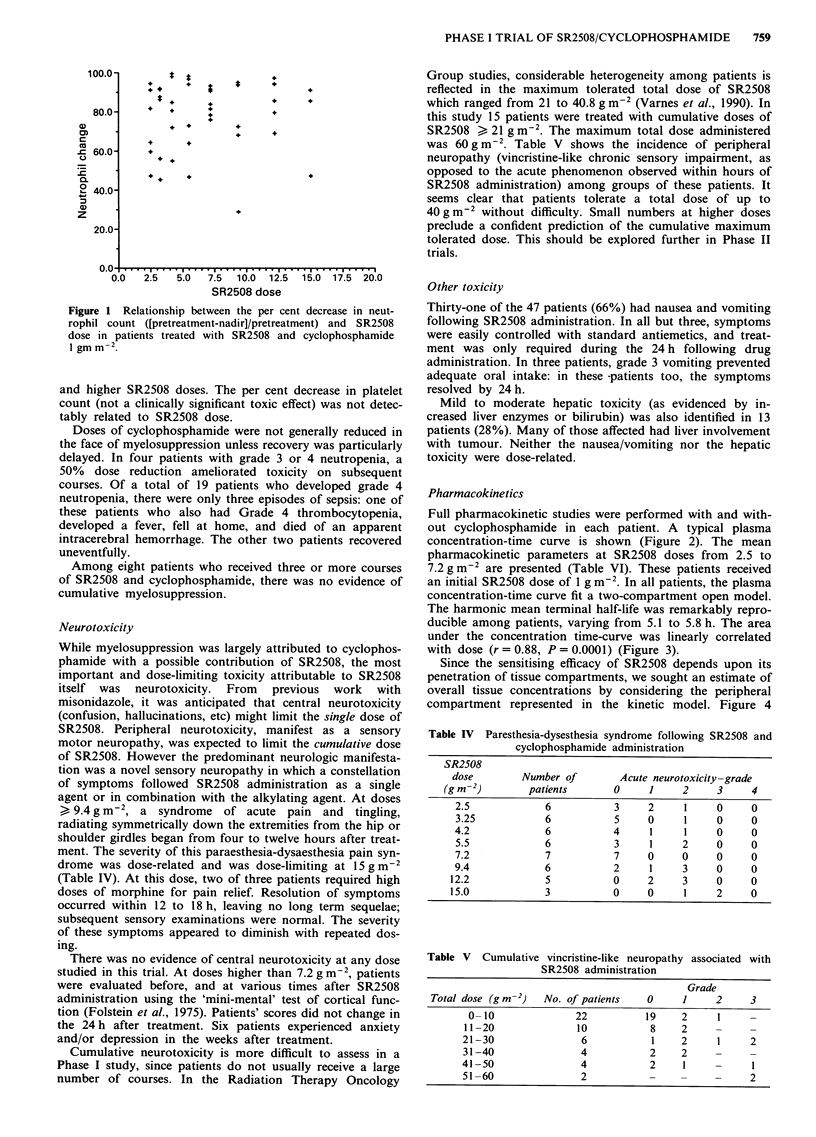

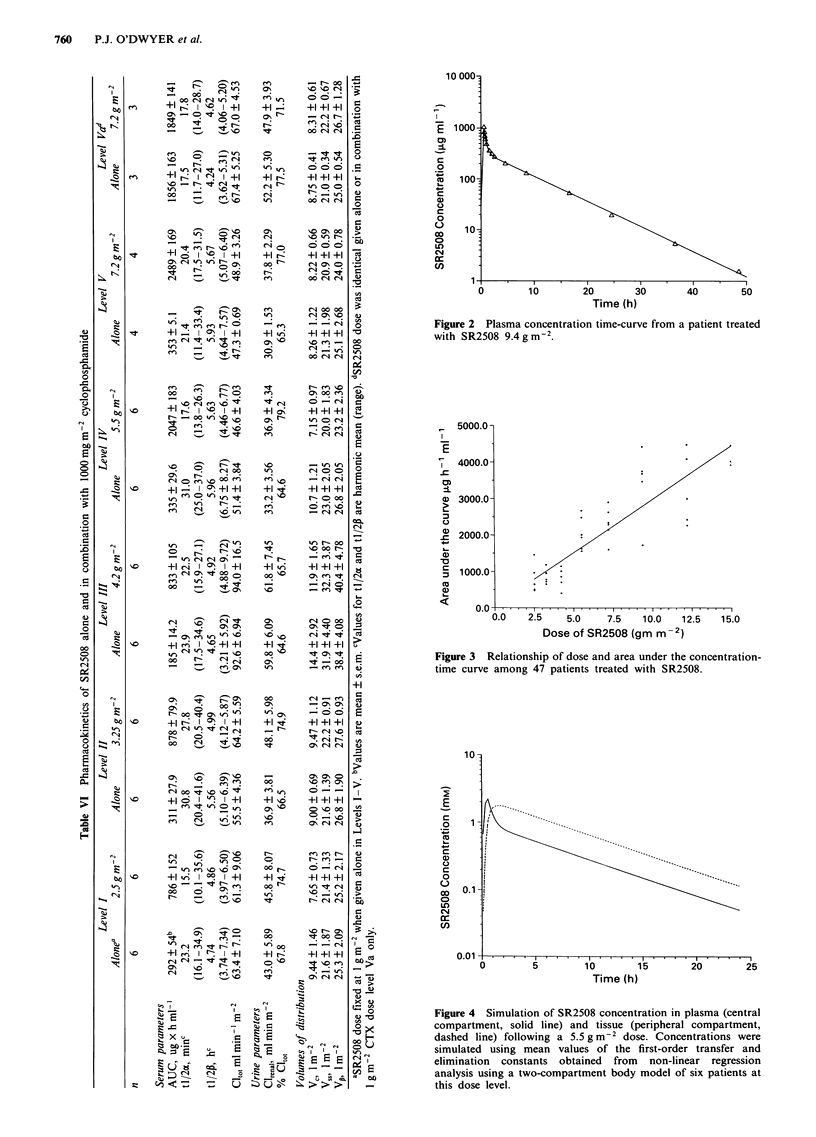

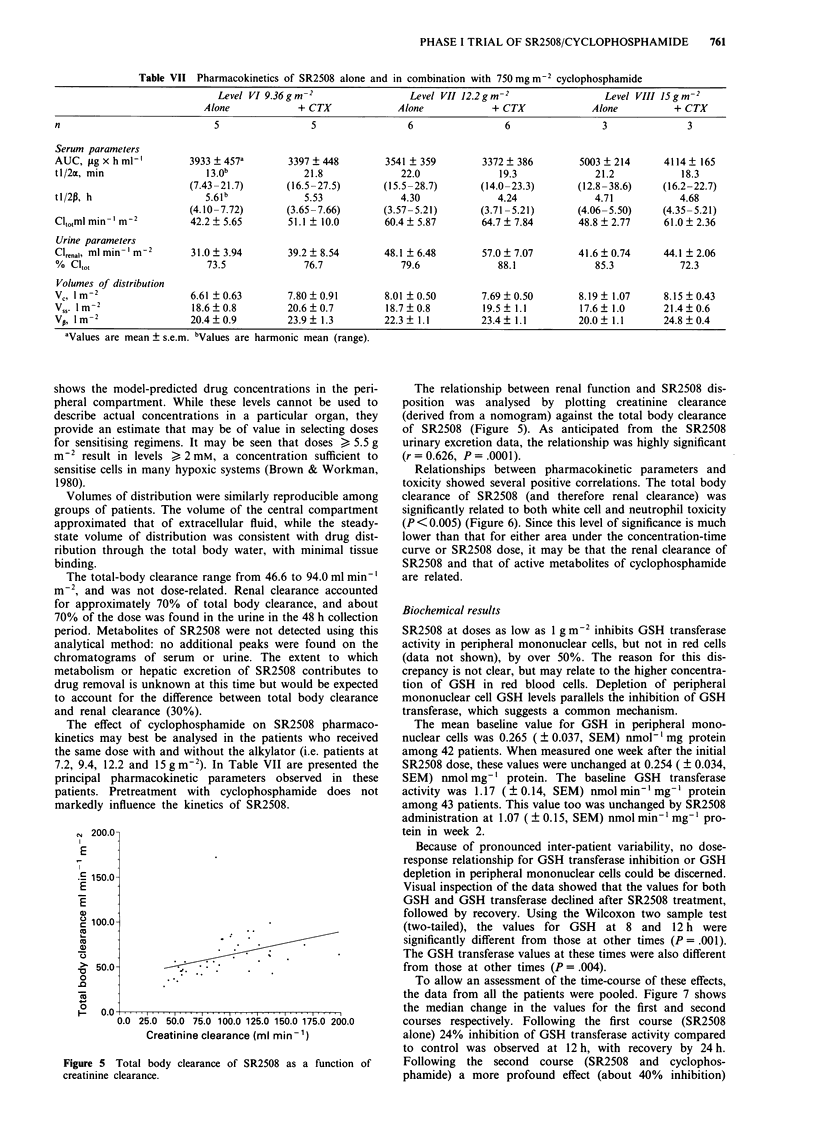

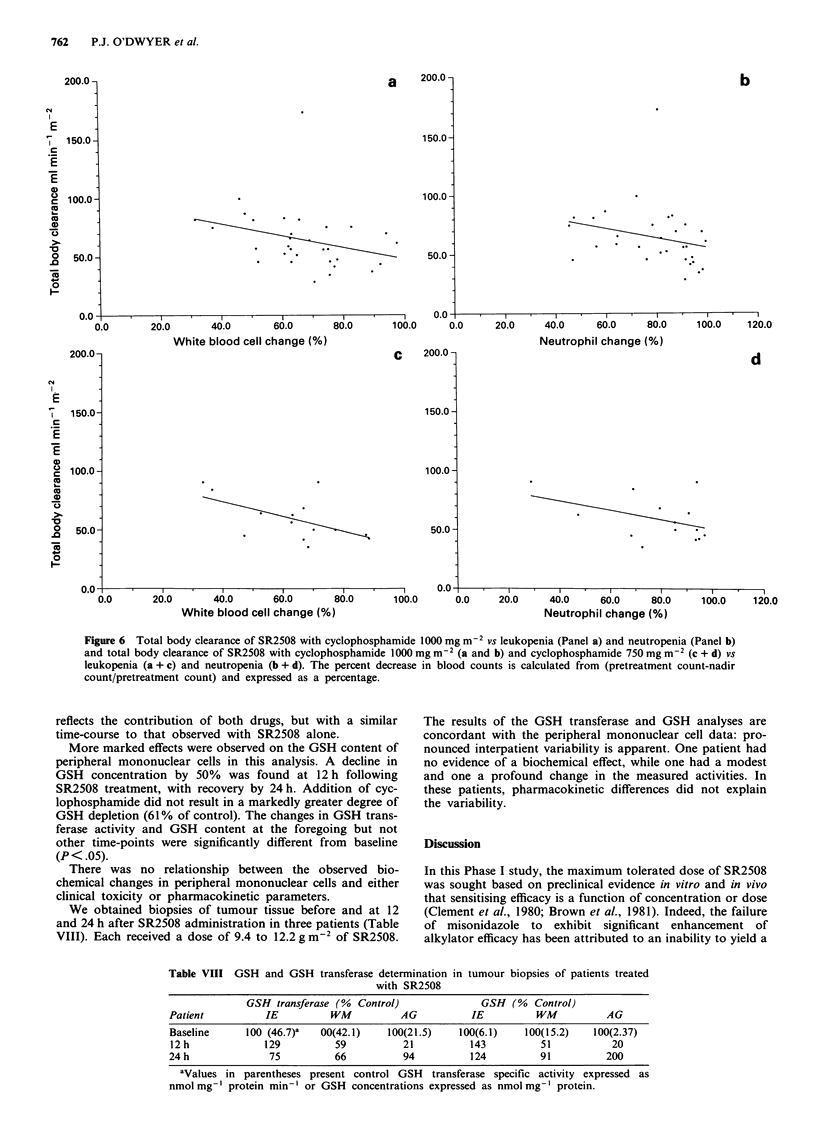

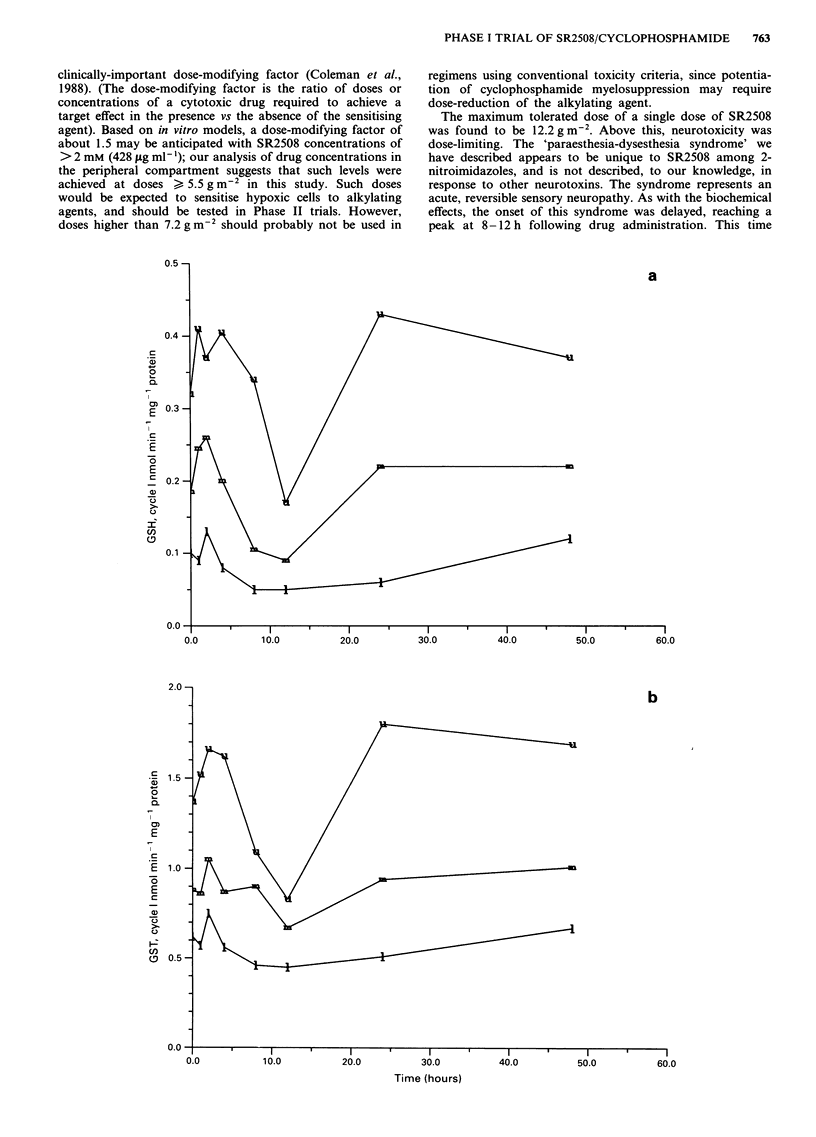

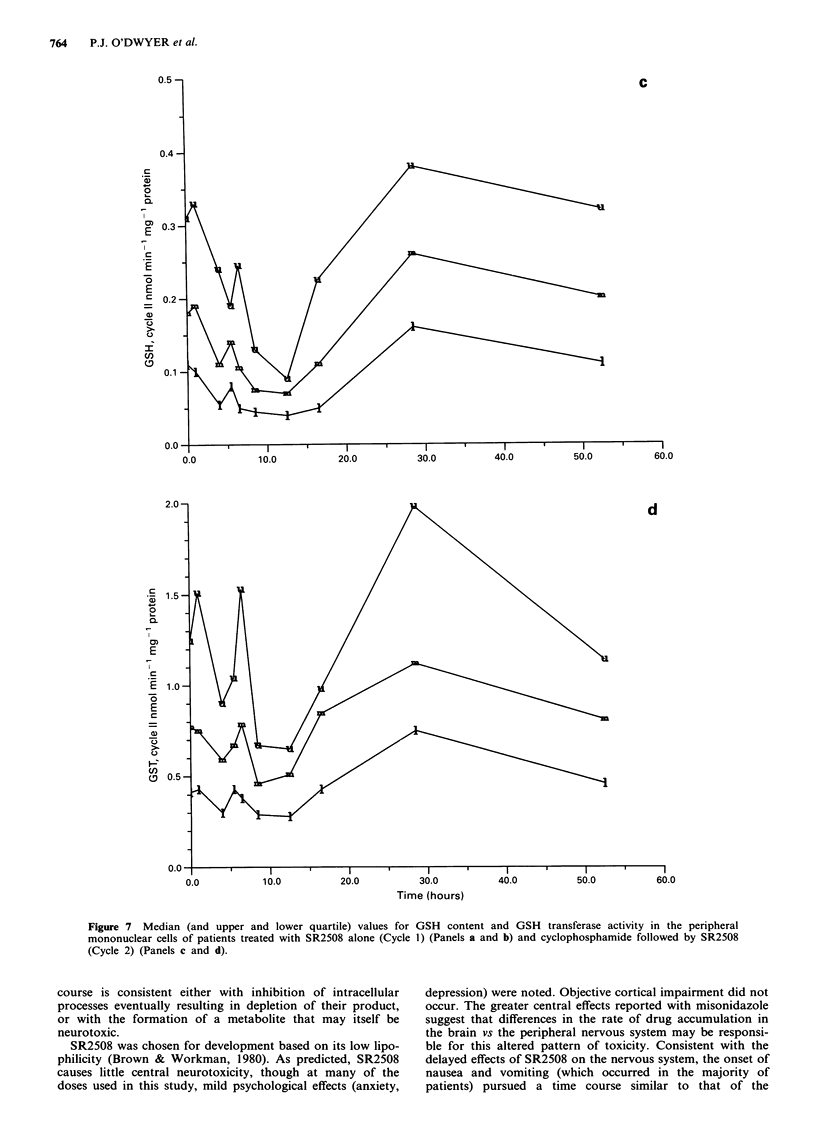

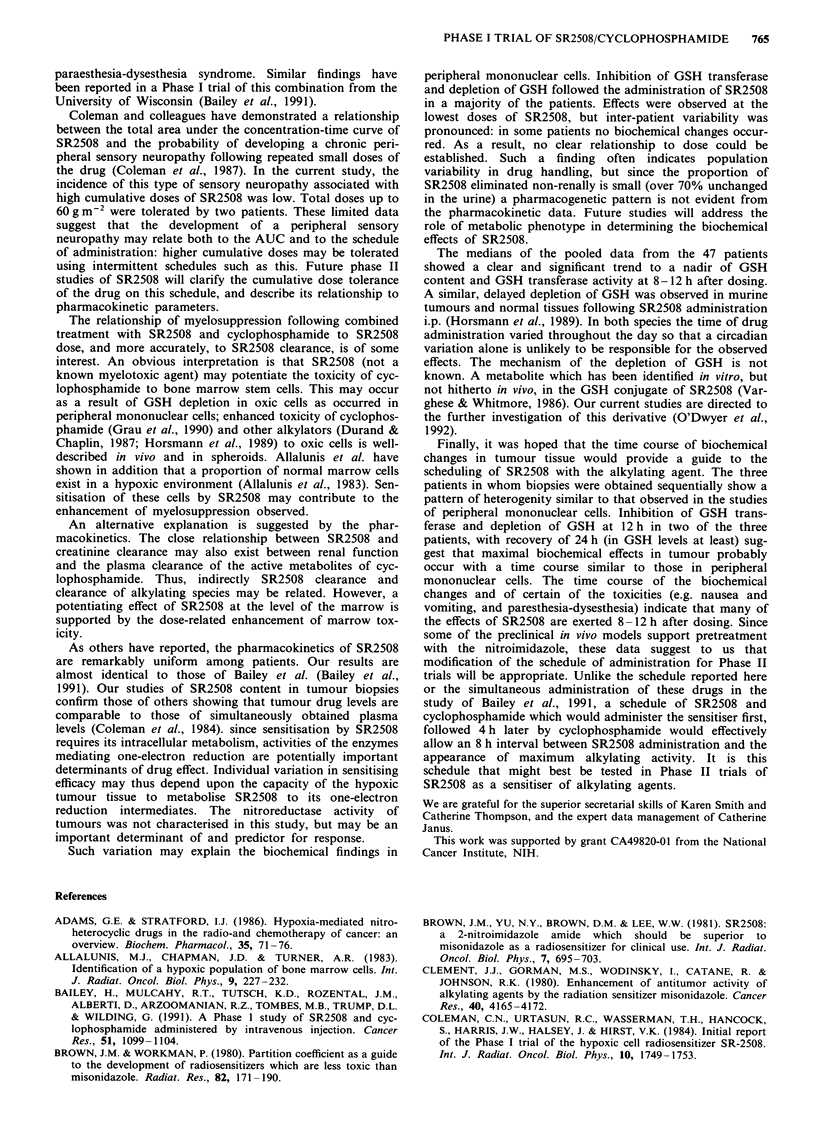

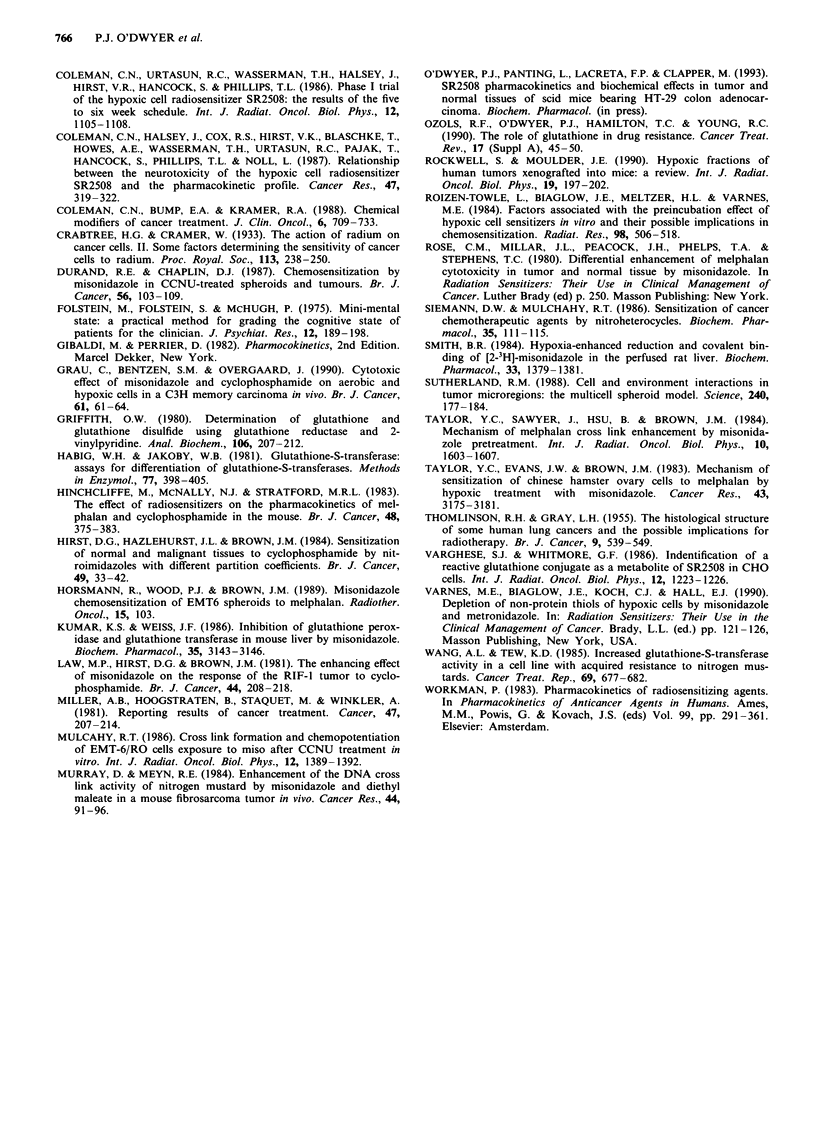


## References

[OCR_01759] Adams G. E., Stratford I. J. (1986). Hypoxia-mediated nitro-heterocyclic drugs in the radio- and chemotherapy of cancer. An overview.. Biochem Pharmacol.

[OCR_01764] Allalunis M. J., Chapman J. D., Turner A. R. (1983). Identification of a hypoxic population of bone marrow cells.. Int J Radiat Oncol Biol Phys.

[OCR_01769] Bailey H., Mulcahy R. T., Tutsch K. D., Rozental J. M., Alberti D., Arzoomanian R. Z., Tombes M. B., Trump D. L., Wilding G. (1991). A phase I study of SR-2508 and cyclophosphamide administered by intravenous injection.. Cancer Res.

[OCR_01776] Brown J. M., Workman P. (1980). Partition coefficient as a guide to the development of radiosensitizers which are less toxic than misonidazole.. Radiat Res.

[OCR_01781] Brown J. M., Yu N. Y., Brown D. M., Lee W. W. (1981). SR-2508: a 2-nitroimidazole amide which should be superior to misonidazole as a radiosensitizer for clinical use.. Int J Radiat Oncol Biol Phys.

[OCR_01787] Clement J. J., Gorman M. S., Wodinsky I., Catane R., Johnson R. K. (1980). Enhancement of antitumor activity of alkylating agents by the radiation sensitizer misonidazole.. Cancer Res.

[OCR_01816] Coleman C. N., Bump E. A., Kramer R. A. (1988). Chemical modifiers of cancer treatment.. J Clin Oncol.

[OCR_01808] Coleman C. N., Halsey J., Cox R. S., Hirst V. K., Blaschke T., Howes A. E., Wasserman T. H., Urtasun R. C., Pajak T., Hancock S. (1987). Relationship between the neurotoxicity of the hypoxic cell radiosensitizer SR 2508 and the pharmacokinetic profile.. Cancer Res.

[OCR_01793] Coleman C. N., Urtasun R. C., Wasserman T. H., Hancock S., Harris J. W., Halsey J., Hirst V. K. (1984). Initial report of the phase I trial of the hypoxic cell radiosensitizer SR-2508.. Int J Radiat Oncol Biol Phys.

[OCR_01801] Coleman C. N., Wasserman T. H., Urtasun R. C., Halsey J., Hirst V. K., Hancock S., Phillips T. L. (1986). Phase I trial of the hypoxic cell radiosensitizer SR-2508: the results of the five to six week drug schedule.. Int J Radiat Oncol Biol Phys.

[OCR_01825] Durand R. E., Chaplin D. J. (1987). Chemosensitization by misonidazole in CCNU-treated spheroids and tumours.. Br J Cancer.

[OCR_01830] Folstein M. F., Folstein S. E., McHugh P. R. (1975). "Mini-mental state". A practical method for grading the cognitive state of patients for the clinician.. J Psychiatr Res.

[OCR_01839] Grau C., Bentzen S. M., Overgaard J. (1990). Cytotoxic effect of misonidazole and cyclophosphamide on aerobic and hypoxic cells in a C3H mammary carcinoma in vivo.. Br J Cancer.

[OCR_01845] Griffith O. W. (1980). Determination of glutathione and glutathione disulfide using glutathione reductase and 2-vinylpyridine.. Anal Biochem.

[OCR_01850] Habig W. H., Jakoby W. B. (1981). Assays for differentiation of glutathione S-transferases.. Methods Enzymol.

[OCR_01855] Hinchliffe M., McNally N. J., Stratford M. R. (1983). The effect of radiosensitizers on the pharmacokinetics of melphalan and cyclophosphamide in the mouse.. Br J Cancer.

[OCR_01861] Hirst D. G., Hazlehurst J. L., Brown J. M. (1984). Sensitization of normal and malignant tissue to cyclophosphamide by nitroimidazoles with different partition coefficients.. Br J Cancer.

[OCR_01867] Horsman M. R., Wood P. J., Brown J. M. (1989). Misonidazole chemosensitization of EMT6 spheroids to melphalan.. Radiother Oncol.

[OCR_01872] Kumar K. S., Weiss J. F. (1986). Inhibition of glutathione peroxidase and glutathione transferase in mouse liver by misonidazole.. Biochem Pharmacol.

[OCR_01877] Law M. P., Hirst D. G., Brown J. M. (1981). Enhancing effect of misonidazole on the response of the RIF-1 tumour to cyclophosphamide.. Br J Cancer.

[OCR_01882] Miller A. B., Hoogstraten B., Staquet M., Winkler A. (1981). Reporting results of cancer treatment.. Cancer.

[OCR_01887] Mulcahy R. T. (1986). Cross-link formation and chemopotentiation of EMT-6/Ro cells exposed to MISO after CCNU treatment in vitro.. Int J Radiat Oncol Biol Phys.

[OCR_01892] Murray D., Meyn R. E. (1984). Enhancement of the DNA cross-linking activity of nitrogen mustard by misonidazole and diethyl maleate in a mouse fibrosarcoma tumor in vivo.. Cancer Res.

[OCR_01904] Ozols R. F., O'Dwyer P. J., Hamilton T. C., Young R. C. (1990). The role of glutathione in drug resistance.. Cancer Treat Rev.

[OCR_01909] Rockwell S., Moulder J. E. (1990). Hypoxic fractions of human tumors xenografted into mice: a review.. Int J Radiat Oncol Biol Phys.

[OCR_01914] Roizin-Towle L., Biaglow J. E., Meltzer H. L., Varnes M. E. (1984). Factors associated with the preincubation effect of hypoxic cell sensitizers in vitro and their possible implications in chemosensitization.. Radiat Res.

[OCR_01926] Siemann D. W., Mulcahy R. T. (1986). Sensitization of cancer chemotherapeutic agents by nitroheterocyclics.. Biochem Pharmacol.

[OCR_01931] Smith B. R. (1984). Hypoxia-enhanced reduction and covalent binding of [2-3H]misonidazole in the perfused rat liver.. Biochem Pharmacol.

[OCR_01936] Sutherland R. M. (1988). Cell and environment interactions in tumor microregions: the multicell spheroid model.. Science.

[OCR_01953] THOMLINSON R. H., GRAY L. H. (1955). The histological structure of some human lung cancers and the possible implications for radiotherapy.. Br J Cancer.

[OCR_01947] Taylor Y. C., Evans J. W., Brown J. M. (1983). Mechanism of sensitization of Chinese hamster ovary cells to melphalan by hypoxic treatment with misonidazole.. Cancer Res.

[OCR_01941] Taylor Y. C., Sawyer J. M., Hsu B., Brown J. M. (1984). Mechanism of melphalan crosslink enhancement by misonidazole pretreatment.. Int J Radiat Oncol Biol Phys.

[OCR_01958] Varghese A. J., Whitmore G. F. (1986). Identification of a reactive glutathione conjugate as a metabolite of SR-2508 in CHO cells.. Int J Radiat Oncol Biol Phys.

[OCR_01970] Wang A. L., Tew K. D. (1985). Increased glutathione-S-transferase activity in a cell line with acquired resistance to nitrogen mustards.. Cancer Treat Rep.

